# Differential Infectivity by the Oral Route of *Trypanosoma cruzi* Lineages Derived from Y Strain

**DOI:** 10.1371/journal.pntd.0001804

**Published:** 2012-10-04

**Authors:** Cristian Cortez, Rafael M. Martins, Renan M. Alves, Richard C. Silva, Luciana C. Bilches, Silene Macedo, Vanessa D. Atayde, Silvia Y. Kawashita, Marcelo R. S. Briones, Nobuko Yoshida

**Affiliations:** 1 Departamento de Microbiologia, Imunologia e Parasitologia, Universidade Federal de São Paulo, São Paulo, Brazil; 2 Unité de Biologie des Interactions Hôte-Parasite, Institut Pasteur, Paris, France; 3 McGill University Health Centre, Montréal, Quebec, Canada; 4 Superintendência da Polícia Técnico-Científica, São Paulo, Brazil; Instituto Oswaldo Cruz, Fiocruz, Brazil

## Abstract

**Background:**

Diversity of *T. cruzi* strains is a central problem in Chagas disease research because of its correlation with the wide range of clinical manifestations and the biogeographical parasite distribution. The role played by parasite microdiversity in Chagas disease epidemiology is still debatable. Also awaits clarification whether such diversity is associated with the outcome of oral *T. cruzi* infection, responsible for frequent outbreaks of acute Chagas disease.

**Methods and Findings:**

We addressed the impact of microdiversity in oral *T. cruzi* infection, by comparative analysis of two strains, Y30 and Y82, both derived from Y strain, a widely used experimental model. Network genealogies of four nuclear genes (SSU rDNA, actin, DHFR-TS, EF1α) revealed that Y30 is closely related to Discrete Typing Unit TcII while Y82 is more closely related to TcVI, a group containing hybrid strains. Nevertheless, excepting one A-G transition at position 1463, Y30 and Y82 SSU rDNAs were identical. Y82 strain, expressing the surface molecule gp82, infected mice orally more efficiently than Y30, which expresses a related gp30 molecule. Both molecules are involved in lysosome exocytosis-dependent host cell invasion, but exhibit differential gastric mucin-binding capacity, a property critical for parasite migration toward the gastric mucosal epithelium. Upon oral infection of mice, the number of Y30 and Y82 parasites in gastric epithelial cells differed widely.

**Conclusions:**

We conclude that metacyclic forms of gp82-expressing Y82 strain, closely related to TcVI, are better adapted than Y30 strain (TcII) to traverse the stomach mucous layer and establish oral route infection. The efficiency to infect target cell is the same because gp82 and gp30 strains have similar invasion-promoting properties. Unknown is whether differences in Y30 and Y82 are natural parasite adaptations or a product of lab-induced evolution by differential selection along the 60 years elapsed since the Y strain isolation.

## Author Summary

Globalization of Chagas disease, from Latin America toward non endemic countries, has become a world health problem. In endemic countries, acute cases of Chagas disease transmitted by oral *Trypanosoma cruzi* infection, have been frequently reported in recent years. The diverse clinical manifestations of the disease are mainly attributed to the highly complex population structure of the parasite. We aimed in this study to investigate the impact of *T. cruzi* microdiversity in oral infection, by comparative analysis of Y30 and Y82 strains, both derived from Y strain, a widely used experimental model. Network phylogenies were inferred to determine their haplotype distribution and classification. Y30 and Y82 were closely related to Discrete Typing Unit TcII and TcVI, respectively. Y82, expressing the surface molecule gp82, was more efficient than Y30, expressing a related gp30 molecule, in establishing infection in mice by oral route. Both molecules are involved in host cell invasion, but exhibit differential gastric mucin-binding capacity, which is critical for parasite migration toward the gastric mucosal epithelium. The number of Y30 and Y82 parasites in gastric epithelial cells differed widely. Our results indicate that gp82-expressing strains are better adapted than gp30-expressing to traverse the stomach mucous layer and establish oral route infection.

## Introduction

Chagas disease, which was formerly restricted to Latin America, has now become a world health problem because of human migration from countries where the disease is endemic to non-endemic countries [1], [2]. The causative agent, *Trypanosoma cruzi*, is distributed in genetically heterogeneous populations with diverse ecological and epidemiological characteristics, and the variability of outcomes observed in *T. cruzi* infections ranges from indeterminate to severe effects to the heart and gastrointestinal tract. In addition to the genetic background and the immunological status of the host, the number of parasite exposures, routes of infection, dose of infectious challenges, it is thought that an important contribution for the diversity in clinical manifestations comes from the highly complex population structure of the parasite and even mixed, multi-strain, infections within an individual host [3]–[8]. Using nine polymorphic microsatellite markers across 211 clones from eight mammals from three different sylvatic foci in South America, Llewellyn et al. [8] defined 49 distinct multilocus genotypes, with as many as 10 isolated from the same host. According to a new consensus for *T. cruzi* intraspecific nomenclature established in 2009, the known isolates and strains should be assigned to one of the six genetic groups or discrete typing units (DTUs), TcI to TcVI [9]. TcI, TcIII and TcIV are the most common in wild transmission cycles [10], [11]. Among domestic transmission cycles, TcI predominates in northern South America, possibly associated with less severe human disease, while TcII, TcV and TcVI are more common in the Southern Cone, where Chagasic megasyndromes are more frequent [11].

Oral infection by *T. cruzi* has been responsible for frequent outbreaks of acute cases of Chagas' disease in different geographical regions in recent years [12]–[15]. The genetic profile of *T. cruzi* in the referred cases has not been determined. Presumably, TcI was implicated in 2007 outbreak of acute Chagas' disease in Caracas, Venezuela [14], where this genetic group prevails. All strains isolated from humans following the 2005 outbreak in the southern state of Santa Catarina, Brazil, belonged to TcII, but strains isolated from triatomines revealed a mixed TcI/TcII profile [16]. Studies of oral *T. cruzi* infection in the murine model have shown that the outcome of infection by TcI, TcII and TcVI strains may vary considerably. Upon oral administration into mice (Placental), metacyclic trypomastigotes of G strain (TcI), isolated from an opossum (Marsupial) in the Brazilian Amazon, were barely detectable in the gastric mucosal epithelium, which is the unique portal of entry toward systemic infection, and this is consistent with the proposed hypothesis of co-evolution of different lineages of *T. cruzi* with Marsupials and Placentals [17]. This low infective capacity was associated with the expression of high levels of pepsin-resistant gp90 [18], the surface molecule that functions as a negative modulator of cell invasion [19]. Metacyclic forms of *T. cruzi* strain SC (TcII), isolated from a patient with acute Chagas' disease, infected after ingesting contaminated sugar cane juice, were found to be highly infective when given orally into mice. These parasites produced high parasitemias and high mortality, although they expressed gp90 at high levels and invaded human epithelial cells in vitro very poorly [18]. This apparent paradox was clarified by the finding that SC parasites recovered from the mouse stomach 1 h after oral administration did not express gp90, which had been completely digested by the gastric juice [18]. Distinct from G and SC strains, metacyclic trypomastigotes of CL strain (TcVI), which expressed a pepsin-resistant gp90 at low levels, efficiently infected mice by the oral route, and replicating parasites were detected in the gastric mucosal epithelium 4 days post-infection [20]. Common to these strains was the expression of gp82, the surface molecule that promotes host cell invasion [18].

Recently, we found that metacyclic forms of two *T. cruzi* samples, identified both as Y strain (TcII), differ in expression profiles of surface molecules and the ability to infect mice by the oral route. One of these isolates, named Y82, expressed gp82 on its surface while the other, named Y30, expressed only gp30, a related glycoprotein. Strain Y is extensively used as *in vitro* and *in vivo* experimental model by several research groups. To determine whether the ‘Y’ strains with distinctive characteristics are variants within the same DTU or belong to different DTUs, we characterized the genetic diversity of these ‘Y’ strains, and characterized their infective properties *in vitro* and in oral infection in mice.

## Methods

### Phylogenetic inference and *T. cruzi* typing

Network phylogenies, or genealogies in the context of intraspecific samples, were inferred to determine the haplotype distribution and classification of *T. cruzi* strains based on gene sequences of small subunit ribosomal DNA (SSU rDNA), actin, dihydrofolate reductase-thymidylate synthetase (DHFR-TS) and elongation factor 1 alfa (EF1 alpha). These sequences were amplified by PCR from total DNA and cloned as described elsewhere [21]. To enable the identification of different haplotypes, PCR products were sequenced directly (Genbank submission ID: 1489955). The Genbank accession numbers for the nucleotide sequences are: TCRUZI-Y82R-ACT (JN942592), TCRUZI-Y82-ACT (JN942593), TCRUZI-YUSP-ACT (JN942594), TCRUZI-YUSPR-ACT (JN942595), TCRUZI-Y30R-ACT (JN942596), TCRUZI-Y30-ACT (JN942597), TCRUZI-YUSP-DHFR-TS (JN942598), TCRUZI-YUSPR-DHFR-TS (JN942599), TCRUZI-Y30-DHFR-TS (JN942600), TCRUZI-Y30R-DHFR-TS (JN942601), TCRUZI-Y82-DHFR-TS (JN942602), TCRUZI-Y82R-DHFR-TS (JN942603), TCRUZI-Y82-EF1 (JN942604), TCRUZI-Y82R-EF1 (JN942605), TCRUZI-YUSP-EF1 (JN942606), TCRUZI-YUSPR-EF1 (JN942607), TCRUZI-Y30-EF1 (JN942608), TCRUZI-Y30R-EF1 (JN942609), TCRUZI-Y30-SSURRNA (JN942610), TCRUZI-Y82-SSURRNA (JN942611). All contig assemblies were finished with error estimates <10^4^ (phred score = 40) as obtained using phred-phrap-consed programs [22], these sequences were added to alignments of Tomazi et al. [21] using Seaview [23] for manual alignments and these alignments were used as infiles for SplitsTree 4 [24]. Parameters for network inference were obtained by modeltest as implemented in Jmodeltest using Akaike Information Criterion on maximum likelihood estimates [25], [26]. Parameters for SSU rDNA were: Model selected: -lnL = 3013.1147, K = 6, AIC = 6038.2295, fA = 0.2482, fC = 0.2219, fG = 0.2630, fT = 0.2670, rAC = 1.0000, rAG = 1.2600, rAT = 1.0000, rCG = 1.0000, rCT = 4.6841, rGT = 1.0000, p(Invariants) = 0.9270, alpha(Gamma) = Equal rates for all sites. For actin tree the parameters were: fA = 0.2475, fC = 0.2484, fG = 0.2662, fT = 0.2379, rAC = 2.8090, rAG = 8.7451, rAT = 1.0000, rCG = 2.8090, rCT = 21.7880, rGT = 1.0000, p(Invariants) = 0.0529, alpha(Gamma) = 0.0110, -lnL = 2032.2651 . For DHFR-TS tree the parameters were: -lnL = 2549.7247, K = 64, fA = 0.2115, fC = 0.2789, fG = 0.3002, fT = 0.2094, rAC = 0.2542, rAG = 1.3989, rAT = 0.1026, rCG = 0.0391, rCT = 2.6514, rGT = 1.0000, p(Invariants) = 0.9230, alpha(Gamma) = 1.1350. Bootstrap frequencies of clades were obtained from 1000 pseudoreplicates. The parameters for EF1alpha networks are in the figure legend for Fig. S1.

### Isolation of a cDNA clone containing a putative gp30 sequence

Complementary DNA from Y30 strain metacyclic trypomastigotes (1×10^8^) was obtained using the AccessQuick™ RT-PCR System (Promega) on total RNA extracted by TRIzol (Invitrogen). Following cDNA synthesis, amplification of the putative *gp30* sequence was performed, based on the presumed similarity between *gp30* and *gp82* sequences. The forward primer 5′-GGATCCATGTTCGTCAGCAGCCTGCTG-3′ corresponded to a sequence that precedes the epitope for mAb 3F6 and contained ATG plus an artificial Bam HI site; the reverse primer 5′-GAATTCGTTCAGTGGGCGGTTGTACAAGAAGA-3′ corresponded to a sequence that follows the highly conserved VTVKNVFLYNR motif, characteristic of all members of the gp85/trans-sialidade superfamily, and contained a stop codon plus an artificial Eco RI site. The sites were added for cloning into pGEX. A total of 40 cycles of denaturing, annealing and elongation at 94°C for 20 sec, 55°C for 30 sec and 72°C for 1 min, respectively, were performed. After purification, using PureLink kit (Invitrogen) the PCR product was cloned in the plasmid vector pGEM-T Easy (Promega). Following ligation to the vector, the product was transformed in *Escherichia coli* strain DH5α, and the colonies were grown in LB broth. Clones containing the expected 760 bp fragment after restriction analysis with Eco RI and Bam HI were sequenced using ABI 3130XL Genetic Analyzer and BigDye Terminator v3.1 (Applied Biosystems).

### Production and purification of the recombinant proteins D21 and J18

The recombinant protein D21, containing the putative gp30 sequence of Y30 metacyclic trypomastigote in frame with gluthatione S-transferase (GST), was generated by subcloning the Eco RI and Bam HI fragment from pGEM-T Easy clones into pGEX-4T3 (Amersham), and subsequent transformation of *E. coli* BL21(DE3). All steps for induction of the recombinant protein D21 and purification of the recombinant protein were essentially the same as previously described for J18, the recombinant protein containing the full length gp82 fused to GST [27]. The amount of purified protein was quantified with Coomassie Plus (Pierce) in 96-well plates, and reading at 620 nm.

### Parasite and host cell invasion assay

*T. cruzi* strains ‘Y’, herein designated Y82 and Y30, were used. Parasites were maintained cyclically in mice and in liver infusion tryptose medium. After one passage in Grace's medium (Invitrogen) or in TC100 medium (Vitrocell, Brazil), to stimulate differentiation, metacyclic forms were purified by passage through DEAE-cellulose column, as described [28]. HeLa cells, the human carcinoma-derived epithelial cells, were grown at 37°C in Dulbecco's Minimum Essential Medium (DMEM), supplemented with 10% fetal calf serum, streptomycin (100 µg/ml) and penicillin (100 U/ml) in a humidified 5% CO_2_ atmosphere. Cell invasion assays were carried out as detailed elsewhere [29], by seeding the parasites onto each well of 24-well plates containing 13-mm diameter round glass coverslips coated with 1.5×10^5^ HeLa cells. After 1 h incubation with parasites, the duplicate coverslips were fixed in Bouin solution, stained with Giemsa, and sequentially dehydrated in acetone, a graded series of acetone∶xylol (9∶1, 7∶3, 3∶7) and xylol. In alternative assays, after 1 h incubations with metacyclic forms, HeLa cells were washed and further incubated for 24 h in DMEM containing 2% fetal calf serum to allow differentiation of internalized parasites into amastigotes, upon which the cells were fixed and stained as above.

### Assay of parasite migration through gastric mucin layer

Polycarbonate transwell filters (3 µm pores, 6.5 mm diameter, Costar, Cambridge MA) were coated with 50 µl of a preparation containing 10 mg/ml gastric mucin from porcine stomach (type III, Sigma) in distilled water. *T. cruzi* metacyclic forms in 600 µl PBS, were added to the bottom of 24-well plates (10^7^ parasites/well). Thereafter, the mucin-coated transwell filters were placed onto parasite-containing wells, and 100 µl PBS were added to the filter chamber. At different time points of incubation at 37°C, 10 µl were collected from the filter chamber for parasite counting and the volume in this chamber was corrected by adding 10 µl PBS.

### Flow cytometry and indirect immunofluorescence assays

Live metacyclic forms (1×10^7^) were incubated for 1 h on ice with specific antibodies. After washings in PBS and fixation with 4% paraformaldehyde for 30 min, the parasites were processed for flow cytometry as described [27]. Assays with fixed and permeabilized parasites were performed by fixation with 4% paraformaldehyde for 30 min, followed by 30 min treatment with 0.1% saponin in PBS at room temperature before incubation with antibodies. The number of fluorescent parasites was estimated with a Becton Dickinson FACscan cytometer. For microscopic visualization of fluorescent live or permeabilized parasites, we followed the protocol described by Atayde et al. [27]. The procedure to visualize the localization of lysosomes in HeLa cells has been detailed elsewhere [30].

### Assays for binding of D21 or J18 protein to host cell/gastric mucin

Binding of the recombinant protein D21 or J18 to target cells was determined by ELISA. HeLa cells, grown in 96-well microtiter plates, were fixed with 4% paraformaldehyde in PBS for 30 min, washed and blocked with PBS containing 2 mg/ml BSA (PBS/BSA). Following 1 h incubation with the recombinant protein in PBS/BSA, the cells were incubated sequentially with anti-J18 antibodies and peroxidase-conjugated anti-mouse IgG, all diluted in PBS/BSA. The final reaction was revealed by *o*-phenilenediamine and the absorbance at 490 nm read in ELx800™ absorbance microplate reader (BioTek). Binding to gastric mucin was determined by using microtiter plates coated with gastric mucin (10 µg/well). After blocking with PBS/BSA, the recombinant proteins were added and the reaction proceeded as above.

### Exocytosis assay

Semi-confluent monolayers of HeLa cells, grown in 24-well plates in DMEM supplemented with 10% fetal calf serum, without phenol red, were incubated in absence or in the presence of the recombinant protein D21, J18 or GST. After 1 h, the supernatants were collected and the cells were lysed in DMEM containing 1% NP-40, and 30 µl of 1 M sodium acetate pH 4.0 was added to decrease pH. Samples were centrifuged for 5 min at 13,000 g and the supernatants were collected, 20 µl aliquots were diluted with 60 µl citrate buffer and 160 µl of 100 mM 4-nitrophenyl N-acetyl-β-D-glucosaminide (Sigma) were added. After 1 h incubation at 37°C, the reaction was stopped by adding 720 µl of 200 mM sodium borate pH 9.8 and absorbance was measured at 405 nm in a Labsystems Multiskan MS plate reader. Exocytosis was expressed as % of total β-hexosaminidase activity (supernatant+cell extract), according to the formula:
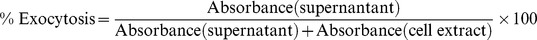



### Oral infection of mice and detection of *T. cruzi* in the gastric epithelium

Four to five week-old female Balb/c mice, bred in the animal facility at Universidade Federal de São Paulo, were used. All procedures and experiments conformed with the regulation of the Universidade Federal de São Paulo Ethical Committee for animal experimentation, in accord with Resolution N° 196 (10/10/1996) of National Council of Health, and the study was approved by the Committee. Oral infection was performed by giving mice *T. cruzi* metacyclic forms (5×10^7^ parasites per mouse), using a plastic tube adapted to a 1 ml syringe. Four days post-infection, the stomach of mice was collected, fixed with 10% neutral formaldehyde for 24 h. After processing by gradual dehydration in a graded series of ethanol solution, followed by xylene immersion and embedding in paraffin, serial 5 µm tissue sections were cut and stained with hematoxylin and eosin.

### Statistical analysis

The significance level of experimental data was calculated using the Student's *t* test, as implemented in the program GraphPad InStat.

## Results

### Molecular network phylogenies of *T. cruzi* strains suggest that Y30 belongs to TcII and Y82 to TcVI, a group of hybrid strains

**Figure 1:**
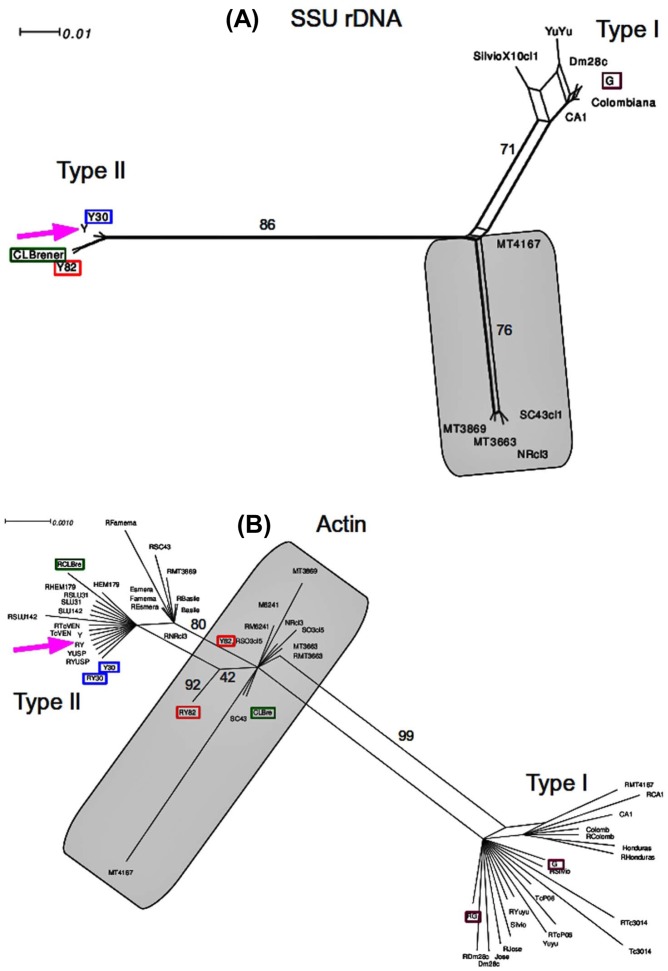
Network genealogy of two *T. cruzi* nuclear gene sequences. Analysis of SSU rDNA (**A**) and actin (**B**) was performed to verify the genetic diversity of Y30 and Y82 strains. Red boxes indicate Y82 haplotypes, blue boxes Y30 haplotypes, purple boxes G (TcI) haplotypes, green boxes CL Brener (TcVI) haplotypes and the pink arrow indicates the Y strain (TcII) cluster. In haplotype names, the suffix R indicates the copy detected by direct sequencing of PCR products from genomic DNA while the other indicates the sequence obtained from cloned PCR products. The shaded area indicates the hybrid zone between the polar TcI and TcII. Numbers above links indicate bootstrap frequencies from 1000 pseudoreplicates. Networks were inferred using SplisTree 4 [24] as described in Material and Methods.

To confirm that Y30 and Y82 strains belonged to distinct genetic groups, network phylogenies were used, because the high degree of hybridization in *T. cruzi* prevents the use of bifurcated trees. The initial inference using SSU rDNA shows that Y30 and Y82 unequivocally cluster with Type II strains (Fig. 1A). Y30 and Y82 differ by one transition A-G in position 1463 while Y30 is identical to Y strain (canonical TcII) and Y82 identical to the type II SSU rDNA of CL Brener, a hybrid TcVI clone. Although for several other markers CL Brener has two haplotypes, it has only the type II SSU rDNA and therefore we checked whether Y30 and Y82 were “pure” TcII, or hybrids like CL Brener. For this, we used datasets already constructed for other publication [21] and also because of the correspondence with findings of Machado and Ayala [31]. The networks of actin gene (Fig. 1B) revealed that both haplotypes of Y30 actin cluster with haplotypes of Y, both from our lab and an additional Y strain from University of São Paulo (courtesy of Dr. Maria Julia Alves and Dr.Walter Colli), as an internal control for among-labs sequence diversity. None of Y82 actin gene haplotypes cluster with “pure” TcII sequences but clustered with the hybrid zone haplotypes (TcIII, TcIV, TcV and TcVI) which are known to be mosaic sequences [32]. Because of the nature of mosaic haplotypes of hybrid strains the bootstraps are considerably lower and the perfect matching with the hybrid DTUs is difficult. DHFR-TS phylogenies confirmed this pattern although with minor variations (Fig. S1A). Both Y30 haplotypes clustered with “pure” TcII haplotypes confirming that Y30 is TcII. Y82 positioning is more debatable. Like CL Brener (TcVI) it has one haplotype clustering with “pure” TcII, although while CL Brener has the other haplotype clearly clustering with TcI, the other haplotype of Y82 is closer to Yuyu (TcI) but also close to the hybrid zone mosaic sequences SC43 (TcV) and Nrcl3 (TcV). Nevertheless, it seems to have a bona fide TcVI pattern. The EF1 alpha network (Fig. S1B) is highly reticulated suggesting, as is the case, intense hybridization, phylogenetic noise and substitution that do not enable unambiguous clusters. Nonetheless, the polarity of Y strain (TcII) and G strain (TcI) is clearly revealed and Y30 haplotypes are closer to TcII while Y82 has a haplotype (Y82R in Fig. S1B) closer to the TcI subnetwork.

### Metacyclic trypomastigotes of Y82 and Y30 strains express and engage related surface molecules to invade host cells

**Figure 2:**
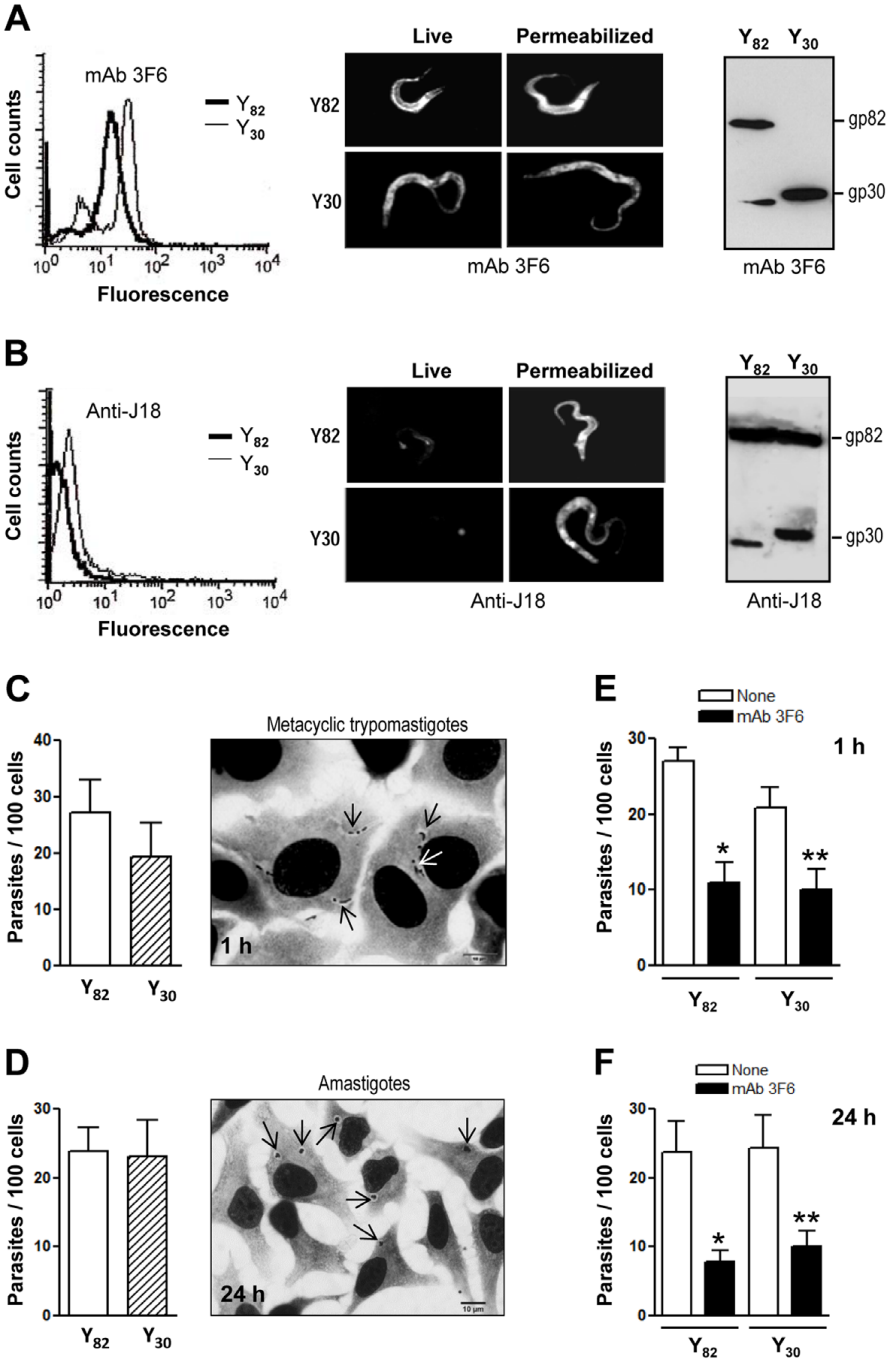
Differential expression of *T. cruzi* Y82 and Y30 strain surface molecules implicated in host cell invasion. Live metacyclic trypomastigotes were incubated for 1 h with mAb 3F6 (A) or polyclonal anti-J18 antibodies (B) and processed for flow cytometry analysis, or for observations at the fluorescence microscope along with paraformaldehyde-fixed saponin-permeabilized parasites. In parallel, detergent-solubilized parasite extracts were analysed by immunoblotting. C) HeLa cells were incubated for 1 h with metacyclic forms, fixed and stained with Giemsa, followed by dehydration as described in experimental procedures. The number of intracellular parasites was counted in a total of 250 cells. The values are the means ± SD of eight independent experiments performed in duplicate. Shown on the right are HeLa cells with internalized metacyclic forms (black arrows), which are surrounded by a clear space, and adherent parasites (white arrow). Scale bar = 10 µm. D) HeLa cells were incubated with metacyclic forms for 1 h, washed to remove non internalized parasites, and incubation proceeded for 24 h in DMEM containing 2% fetal calf serum, upon which the cells were fixed and stained as in (C) for parasite counting. The values are the means ± SD of four independent assays performed in duplicate. Shown on the right are HeLa cells with amastigotes (black arrows). Scale bar = 10 µm. E) HeLa cells were incubated with metacyclic forms, untreated or pretreated with mAb 3F6, and the cells were processed as in (C). The values are the means ± SD of three independent experiments performed in duplicate. The difference between the parasites treated with mAb 3F6 and the untreated control was significant (*p<0.005, **p<0.01). F) Metacyclic forms, untreated or pretreated with mAb 3F6, were incubated for 1 h with HeLa cells, non internalized parasites were washed away, and incubation proceeded for 24 h as in (D). The values are the means ± SD of three independent assays performed in duplicate. MAb 3F6 significantly inhibited parasite invasion (*p<0.005, **p<0.01).

Metacyclic forms of Y82 and Y30 strains were analyzed by flow cytometry, immunofluorescence and Western blot, using mAb 3F6 as well as polyclonal antibodies directed to the recombinant protein J18, which contains the full-length gp82 sequence [33]. MAb 3F6 recognized live parasites of both strains by flow cytometry, reacted in Western blot with major bands identified as gp82 and gp30 in Y82 and Y30 strains, respectively, and revealed an homogeneous distribution of surface gp82 as opposed to a patchy profile of gp30 (Fig. 2A). Polyclonal anti-J18 antibodies, which barely recognized live parasites of either strain, reacted with fixed and saponin-permeabilized parasites (Fig. 2B). Although Y30 metacyclic forms lack the surface gp82 recognized by mAb 3F6, they do express intracellular gp82 molecules that are detectable by anti-J18 antibodies in parasite extracts by Western blot (Fig. 2B). Cell invasion assays were performed by incubating HeLa cells with metacyclic forms for 1 h, followed by fixation, staining with Giemsa and serial dehydration, as described in the methods section. This staining procedure allows a clear discrimination between adherent and internalized parasites (Fig. 2C and Fig. S3A). Despite the higher multiplicity of infection (MOI = 20), the number of internalized Y30 strain parasites was slightly lower as compared to Y82 strain (MOI = 10) (Fig. 2C). To further ascertain the effective internalization of parasites, an additional experiment was performed. Hela cells were incubated with metacyclic forms for 1 h and then washed to remove non internalized parasites. Subsequently, DMEM containing 2% fetal calf serum was added and incubation proceeded for 24 h, upon which the cells were fixed and stained as above. For both strains, the number of intracellular parasites was comparable to the number detected after 1 h incubation (Fig. 2D and Fig. S3B). The similarity in intracellular parasite numbers at 1 h and 24 h was expected because metacyclic forms remain in the parasitophorous vacuole for relatively long time, before reaching the cell cytoplasm and transforming into amastigotes. Assays were also performed to test the effect of mAb 3F6 on host cell invasion by Y82 and Y30 parasites. Pre-incubation of metacyclic forms with mAb 3F6 for 30 min, before 1 h incubation with HeLa cells, diminished the infectivity of both strains (Fig. 2E). In HeLa cells incubated for 1 h with mAb 3F6-treated parasites, followed by washing and 24 h incubation, the number of amastigotes was significantly lower in mAb 3F6-treated cells than in control untreated cells (Fig. 2F). Altogether, these results indicate that gp82 and gp30 are related molecules that are involved in the internalization of Y82 and Y30 strains, respectively.

### The recombinant protein D21 exhibits host cell-binding and lysosome exocytosis-inducing properties

**Figure 3:**
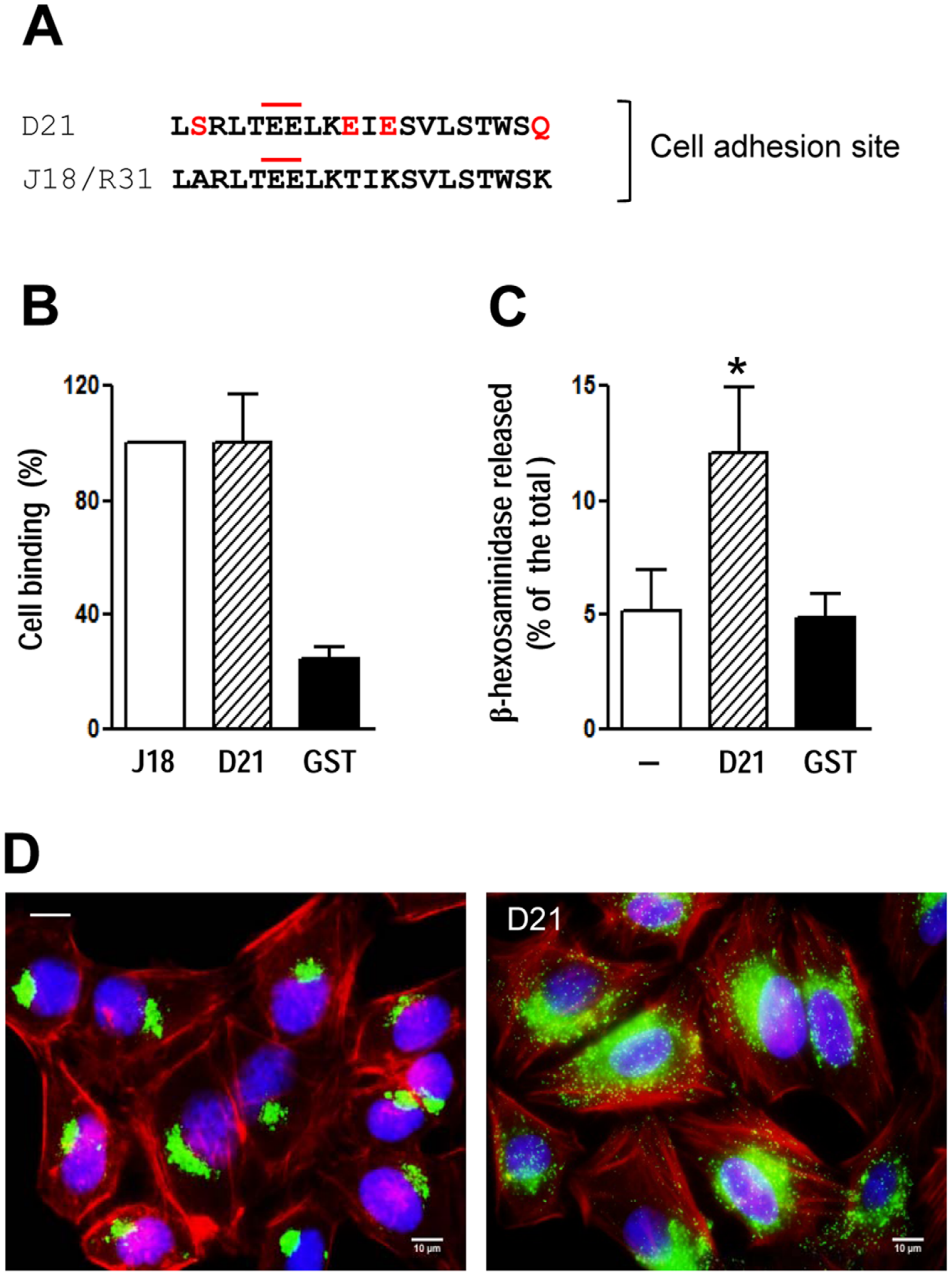
Binding of the recombinant protein D21 to HeLa cells and induction of lysosomal exocytosis. A) D21 sequence was aligned to the J18 sequence previously identified as the host cell binding site, with the changed residues highlighted in red. The pair of contiguous glutamic acid residues implicated in cell binding is marked by an upper red line. B) HeLa cells were incubated with the indicated proteins, at 20 µg/ml, and the binding assay proceeded as described in the methods section. GST was used as control. Values, given relative to the binding of J18, which were fixed to 100, are the means ± SD of three independent assays performed in duplicate. C) Semi-confluent HeLa cell monolayers were incubated in absence or in the presence of D21 at 20 µg/ml. After 1 h, the supernatants were collected and the release of β-hexosaminidase was measured. Exocytosis was expressed as percentage of total β-hexosaminidase activity (supernatant+cell extract). The values are the means ± SD of three independent assays. The intensity of exocytosis was significantly higher in the presence of D21 (*p<0.05). GST had no effect. D) Hela cells were incubated for 1 h in absence or in the presence of D21, and then processed for immunofluorescence using anti-Lamp-2 antibody and Alexa Fluor 488-conjugated anti-mouse IgG (green), phalloidin-TRITC (red) for actin visualization and DAPI (blue) for DNA.

We next sought to obtain further molecular information on gp30. To that end, we cloned different members of the gp82 family from cDNA obtained from Y30 strain metacyclic trypomastigotes, using primers designed to amplify the region between the mAb epitope and the conserved VTVKNVFLY motif. One clone, named D21, GenBank accession number JF745802), displayed 57% and 59% identity, respectively, with the sequences deduced from cDNA from clones J18 (GenBank L14824) and R31 (GenBank AF128843) (Fig. S2), derived respectively from highly divergent G and CL strains and containing gp82 sequences with 97.9% identity [34]. To determine whether the sequence identified as the cell binding site of gp82 [35] was shared by gp30, equivalent sequences of D21 and J18/R31 were compared. D21 exhibited 80% identity with J18/R31 and preserved the two contiguous glutamic acid residues (Fig. 3A), previously shown to be required for cell binding [35]. Recombinant proteins D21 and J18, in fusion with GST, were produced and their ability to bind to HeLa cells was compared. Both proteins bound to HeLa cells in a similar manner whereas GST, used as control, exhibited low binding capacity (Fig. 3B). We also checked whether D21 shared with J18 the property of inducing target cell lysosome exocytosis, an event required for *T. cruzi* invasion [36], [37]. HeLa cells were incubated for 1 h with D21, at 20 µg/ml, and the lysosome enzyme β-hexosaminidase was measured in the culture supernatant and the cell extract. Exocytosis increased significantly in cells incubated with protein D21, as compared to GST (Fig. 3C), in a manner similar to J18 [30]. The D21-induced increase in exocytosis resulted from mobilization of lysosomes from the perinuclear region to the cell periphery (Fig. 3D).

### Metacyclic forms of Y82 and Y30 strains use similar mechanisms to enter host cells

**Figure 4:**
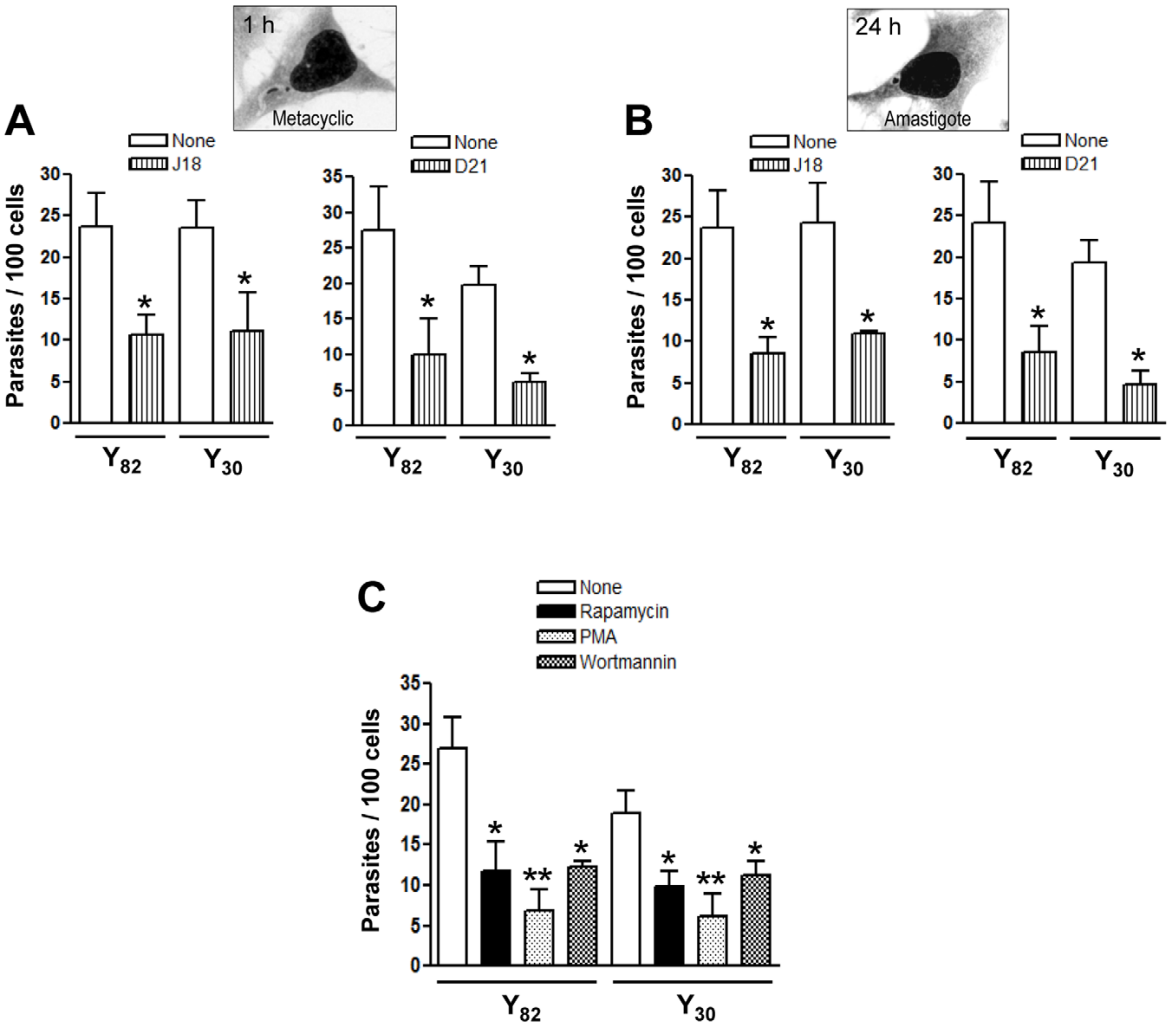
Host cell invasion by Y30 and Y82 strain metacyclic forms through similar mechanisms. A) and B) HeLa cells were incubated with the indicated recombinant protein at 40 µg/ml. After 15 min, metacyclic trypomastigotes were added and incubation proceeded for 1 h before fixation and staining with Giemsa, as in Fig. 1C. The number of internalized parasites was counted in a total of 250 cells. Values are the means ± SD of three independent assays performed in duplicate. The difference between cells treated with the recombinant protein and the untreated control was significant (*p<0.05). C) HeLa cells were preincubated for 30 min with the indicated drug, at 50 nM. After removal of the drug, the parasites were added and the incubation proceeded for 1 h, before processing for parasite counting as above. The values are as in A and B. Parasite invasion in drug-treated cells was significantly reduced (*p<0.05, **p<0.01).

The sequence similarity of the cell binding site between D21 and J18 proteins (Fig. 3A), and their ability to effectively bind to host cells (Figs. 3B), plus the finding that HeLa cell invasion by metacyclic forms of Y82 and Y30 strains was inhibited by mAb 3F6 (Fig. 2D), suggested the possibility that similar mechanisms mediated the internalization of these parasites. A set of experiments was performed to address that question. Metacyclic forms of both strains were incubated with HeLa cells for 1 h, in absence or in the presence of the recombinant protein D21 or J18, at 40 mg/ml. Internalization of both strains was inhibited by D21 or J18 in a similar manner (Figs. 4A and 4B). Inhibition of HeLa cell invasion by both strains was also inhibited by treatment of parasites with antibodies directed to D21 protein (data not shown). To have a clue as to the signaling cascades induced in the host cell during invasion of these parasites, assays were performed with HeLa cells pretreated for 30 min with rapamycin, an inhibitor of mammalian target of rapamycin (mTOR), wortmannin, an inhibitor of the lipid kinase phosphatidyl inositol 3 kinase (PI3K), or PMA, a drug that can downregulate protein kinase C (PKC), all of them at 50 nM. These enzymes have been implicated in the process of gp82-dependent metacyclic trypomastigote invasion [30], [38]. When compared to untreated controls, HeLa cells pretreated with either of these drugs displayed reduced susceptibility to infection by both strains (Fig. 4C).

### Differential capacity of D21 and J18 to bind to gastric mucin correlates with the ability of Y30 and Y82 strains to migrate through the gastric mucin layer

**Figure 5:**
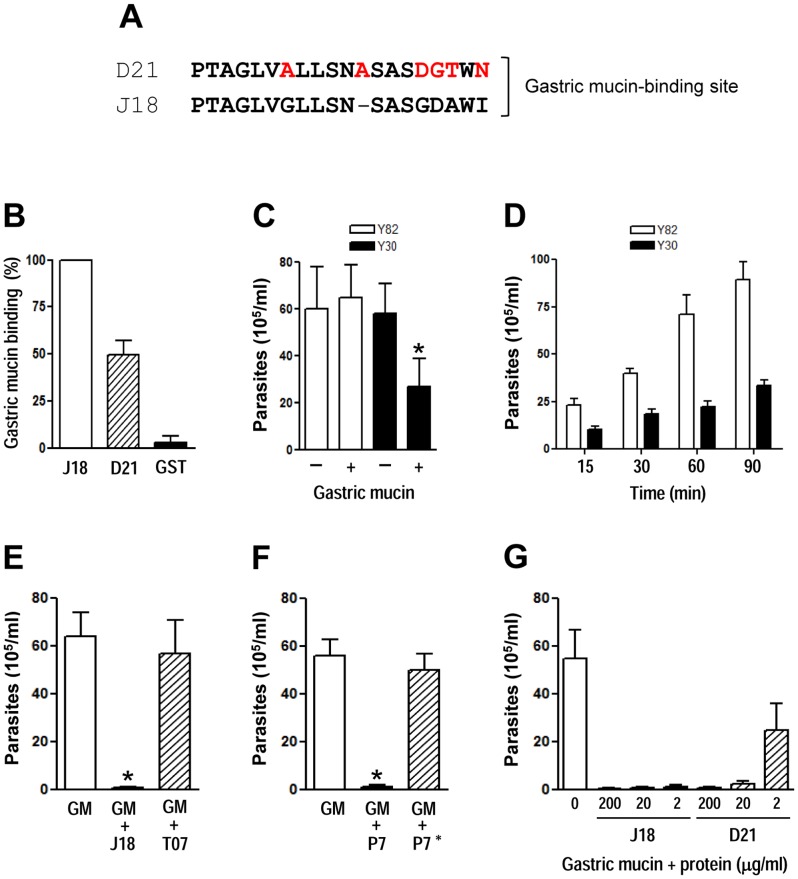
Binding of the recombinant protein D21 to gastric mucin and metacyclic trypomastigote migration. A) D21 sequence was aligned to the J18 sequence previously identified as the gastric mucin binding site, with the changed residues highlighted in red. B) Microtiter plates coated with gastric mucin were incubated with the indicated proteins, at 20 µg/ml, and the assay proceeded as described in the methods section. GST was used as control. Values, given relative to the binding of J18, which were arbitrarily fixed to 100, are the means ± SD of three independent assays performed in duplicate. C) Polycarbonate transwell filters coated or not with gastric mucin were placed onto parasite-containing wells. After 60 min, samples from the filter chamber were collected and the number of parasites counted. Values are the means ± SD of three independent assays. The difference between Y30 migration through non coated and gastric mucin-coated filter was significant (*p<0.05). (D) Transwell filters coated with gastric mucin were placed onto parasite-containing wells, and at different time points, the number of parasites collected in the upper chamber was counted. Values are the means ± SD of four independent experiments. E-G) Transwell filters coated with gastric mucin alone or mixed with the recombinant protein J18 or T07 (E), with synthetic peptide P7 or P7* (F), with the recombinant protein J18 or D21 (G), were placed onto Y82 parasite-containing wells. After 60 min, samples from the filter chamber were collected and the number of parasites counted. Values are the means ± SD of three independent experiments. Significant difference was found between the control and the gastric mucin mixed with J18 or P7 (*p<0.0005).

When compared to the gastric mucin-binding site of J18, the equivalent sequence in D21 exhibited several substitutions. In addition to three conservative substitutions, there was a switch from GD to DG, and an extra alanine residue in D21 (Fig. 5A). To test the ability of D21 to bind to gastric mucin, ELISA assays were performed using microtiter plates coated with gastric mucin (10 µg/well), which were incubated with the recombinant protein D21 or J18 as control, at 20 µg/ml, followed by reaction with anti-GST antibodies. D21 binding to gastric mucin was lower than J18 (Fig. 5B). Essentially the same results were obtained when anti-D21 or anti-J18 antibodies were used. Next, we examined the ability of metacyclic forms to migrate through a gastric mucin layer. First, we checked whether gastric mucin functioned as a barrier to parasite migration. Polycarbonate transwell filters coated or not with gastric mucin (10 mg/ml) were placed onto parasite-containing wells. After 1 h the number of parasites recovered in the upper chamber was counted. Comparable numbers of Y82 strain traversed the uncoated and gastric mucin-coated filters, whereas the significantly fewer Y30 parasites migrated through mucin-coated filters as compared to empty filters (Fig. 5C). Next, migration assays were performed with gastric mucin-coated transwell filters in which parasites were collected from the filter chamber at various time points.. When compared to Y82 strain MT, the number of Y30 strain metacyclic forms that translocated through the gastric mucin layer was much lower (Fig. 5D). To demonstrate that gp82 was implicated in metacyclic trypomastigote migration through the gastric mucin layer, assays were performed with transwell filters coated with gastric mucin (10 mg/ml) alone or mixed with the recombinant protein J18 (1 mg/ml). As control, we also used filters coated with gastric mucin mixed with T07 (1 mg/ml), a recombinant protein containing a sequence of metacyclic stage surface molecule gp90 [39], which shares considerable sequence identity with gp82 but lacks the gastric mucin-binding site identified in gp82. Migration of Y82 metacyclic forms through the gastric mucin mixed with J18 was drastically reduced, whereas the recombinant protein T07 was devoid of inhibitory effect (Fig. 5E). Gastric mucin mixed with GST, to which the recombinant proteins J18 and T07 are fused, allowed efficient parasite migration (data not shown). In addition, we tested the effect of synthetic peptides P7 and P7*. Peptide P7, corresponding to the gp82 gastric mucin binding site, was shown to inhibit the invasion of gastric mucosal epithelium by metacyclic forms, when administered into mice before the parasites [40], presumably because it blocks the parasite migration through the gastric mucus. As shown in Fig. 5F, the presence of P7 profoundly affected the parasite traversal through the gastric mucin. On the other hand, peptide P7*, which has the same composition as P7 but with a scrambled sequence and is devoid of inhibitory effect on oral infection by metacyclic forms [40], had no effect on parasite migration (Fig. 5F). Even at 2 µg/ml, J18 mixed to gastric mucin acted as a potent barrier to parasite migration (Fig. 5G). The effect of recombinant protein D21 mixed to gastric mucin also displayed an inhibitory effect on parasite migrations but to a lower degree than J18 (Fig. 5G). Migration of Y30 metacyclic forms through gastric mucin mixed with J18 was also strongly inhibited (data not shown).

### Host cell invasion of Y30 strain metacyclic forms is impaired by gastric mucin

**Figure 6:**
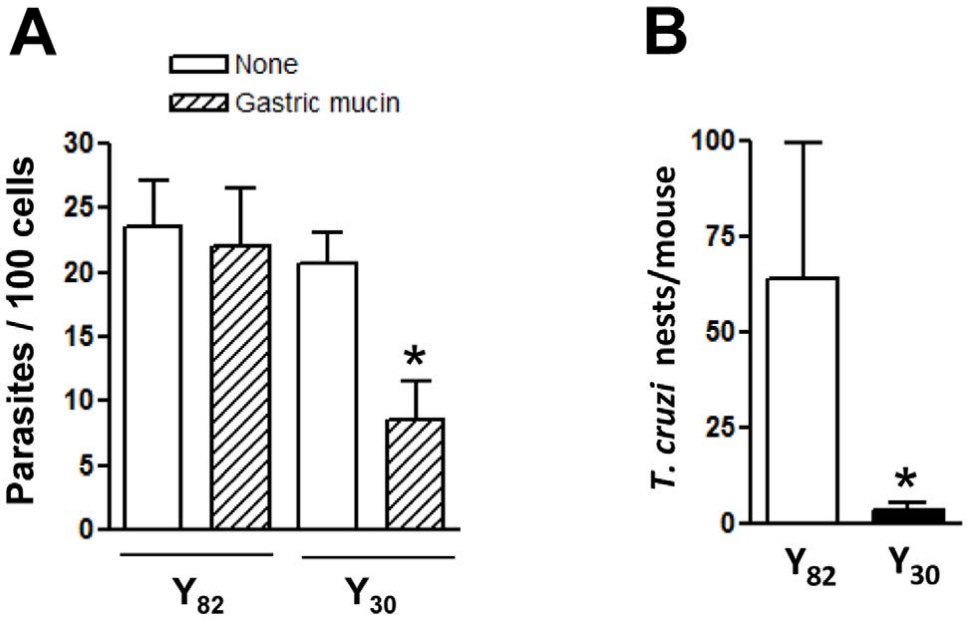
Effect of gastric mucin on host cell invasion by MT of Y82 and Y30 strains in vitro and in vivo. A) HeLa cells were incubated with gastric mucin (20 mg/ml) and then metacyclic forms were added. After 1 h incubation, the cells were fixed and Giemsa-stained for intracellular parasite counting. Values are the means ± SD of four independent assays performed in duplicate. The difference in absence and in the presence of gastric mucin was significant for Y30 strain (*p<0.005). B) Balb/c mice were administered orally with metacyclic forms of Y82 strain (n = 5) or Y30 (n = 5). Four days later, the stomachs of mice were collected and the histological sections were stained by hematoxylin and eosin. The number of amastigote nests was counted in 16–18 equivalent tissue sections of the mouse stomach, and the representative results are shown, with bars corresponding to the variation in the number of parasite nests between sections. The difference in parasite numbers between Y82 and Y30 strains was significant (*p<0.0001).

Since migration of Y30 strain through the mucin layer was reduced, as compared to Y82 strain, we reasoned that these parasites might differ in their ability to invade host cells in the presence of gastric mucin. HeLa cells were incubated with gastric mucin (20 mg/ml) and 30 min later metacyclic forms were added. After 1 h incubation, the number of internalized parasites was counted. As shown in Fig. 6A, invasion of Y30 strain metacyclic forms was reduced in the presence of gastric mucin whereas the infectivity of Y82 strain was not affected. If gastric mucin represents a barrier for Y30 strain MT to reach the underlying target epithelial cells, their infective capacity by the oral route would be lower than that of Y82 strain. Mice were infected orally with MT of both strains and four days later their stomachs were collected and processed for histological preparations and counting of amastigote nests. The number of parasites replicating in the gastric mucosa in Y30 strain-infected mice was much lower, as compared to Y82 strain-infected mice (Fig. 6B).

## Discussion

We have shown that *T. cruzi* strains, identified as “Y” strain and herein designated Y30 and Y82 strains, which are members of distinct genetic groups, exhibit distinct infective properties. While metacyclic forms of Y82 strain efficiently invaded the gastric mucosal epithelium upon oral administration into mice, the rate of invasion by Y30 strain was much lower. Our results suggest that such difference in infectivity is mainly determined by the differential expression of surface molecules.

The metacyclic stage-specific surface molecule gp82, which is highly conserved in *T. cruzi* strains of distinct lineages [41], binds to gastric mucin, which is the main component of the mucus layer that protects the gastric mucosa. Binding to gastric mucin is the first and critical step for the subsequent migration of parasites toward the target cells. Therefore, the selective binding of gp82 to gastric mucin is possibly an important determinant in directing *T. cruzi* metacyclic trypomastigotes to stomach mucosal epithelium. Upon oral administration of insect-derived metacyclic forms into mice, Hoft et al. [42] identified the gastric mucosa as the unique target for *T. cruzi* entry, no parasites being detectable elsewhere within the mucosa of the oropharynx or esophagus. Other pathogenic microorganisms of the gastrointestinal tract also rely on selective binding to mucin molecules as a prerequisite for the establishment of infection. *Helicobacter pylori*, which binds to human gastric mucin, colonizes gastric mucosa [43] and *Shigella*, which binds specifically to human colonic mucin but not to small intestine mucin, invades and multiplies within cells of the colonic epithelium [44]. Metacyclic forms of Y82 strain were found to have the ability to migrate through a gastric mucin and, when administered orally into mice, they invaded gastric mucosal epithelial cells, possibly in gp82-mediated manner, as is the case of invasion of cultured human epithelial cells. Essentially the same results were previously obtained with metacyclic forms of CL strain (TcVI), which efficiently entered epithelial cells in the stomach when given orally into mice [20]. CL strain parasites bound to gastric mucin in gp82-mediated manner, but not to submaxillary mucin, and had their internalization inhibited when submaxillary mucin was present in cell invasion assays, whereas the presence of gastric mucin, even at high concentration, had no effect [40]. The surface profile of Y82 and CL strain metacyclic form is almost indistinguishable [34], both expressing low levels of gp90, the down regulator of host cell invasion [19], and this is reflected in their infective properties in vitro as well as in vivo. Whether what was found in Y82 and CL strains is a general feature of DTU TcVI remains to be established.

As opposed to gp82-expressing TcVI strains, the gp82 deficiency in metacyclic forms of Y30 strain (TcII) impaired their migration through the gastric mucin layer. Although the surface molecule gp30 expressed in Y30 strain is related to gp82, its capacity to bind to gastric mucin is lower, possibly due to the differences in the peptide sequence corresponding to the gastric mucin-binding site. As a consequence, fewer Y30 strain parasites traversed the gastric mucin layer in vitro. The fact that, upon oral administration into mice, fewer Y30 strain parasites were found in the gastric epithelial cells, as compared to Y82 strain, is consistent with the difficulty in overcoming the barrier represented by the mucus layer. Once the target cells are reached, the internalization of Y30 strain may be accomplished in the same manner as Y82 strain, by engaging gp30 that was shown to be involved in host cell invasion in vitro. Like gp82, binding of gp30 to target cells induces lysosome mobilization, leading to exocytosis required for the biogenesis of parasitophorous vacuole during *T. cruzi* invasion [36], [37]. It should be mentioned that a lower efficiency in establishing infection by the oral route has previously been observed in gp82-deficient parasite strains isolated from chronically infected patients, which also expressed a gp30 molecule with reduced gastric mucin-binding capacity [45]. Is the expression of gp30 identified by monoclonal antibody 3F6 a characteristic shared by TcII strains in general? This is a question that awaits further investigation.

The two *T. cruzi* strains, Y30 and Y82, both initially identified as “Y strain”, were here assigned to DTUs TcII and TcVI based on haplotype patterns and phylogenetic relatedness. This strengthens the importance of proper molecular systematics analysis on strain genotypes to avoid underestimates of the genetic diversity which may cause misinterpretation, due to incorrect classification, of comparative experimental data on cell biology, biochemistry and immunology of *T. cruzi*. At first, we presumed that the Y82 and Y30 strains were very closely related and belonged to the same genetic group. Our effort in inferring the phylogenetic position of the two strains allowed us to correctly associate their infective characteristics to proper DTUs. However, with the present data it is still not possible to answer the question of whether the two strains evolved from the same original parasite population, or clone, by differential selection caused by the diverse culturing schemes in different laboratories. The “Y strain” was isolated almost 60 years ago from a patient at the acute phase of Chagas' disease and rendered virulent in mice by serial passages [46]. Distinct strains may have been derived from the original isolate and, as a matter of fact, strain selection by different schedules of mouse passage of an initially mixed infection has been demonstrated [47]. The Y82 strain used in this study has been maintained through alternate passages in mice and liquid culture medium as a counterpart of the insect milieu, to mimic *T. cruzi* life cycle [48], whereas Y30 strain was previously maintained through in vivo passages in non-syngeneic Swiss Webster mice [49]. Besides the natural diversity of *T. cruzi*, researchers have to cope with “lab diversity”, which should not be underestimated, to make sense of experimental data and integrate the results from different labs in general models of *T. cruzi* infection.

## Supporting Information

Figure S1 **Network genealogies of DHFR-TS and EF1 alpha gene sequences.** (**A**) DHFR-TS and (**B**) EF1 alpha. The highly reticulated topology in (B) is indicative of intense hybridization, or other gene homogenizing processes, although not informative for grouping and typing. Red boxes indicate Y82 haplotypes, blue boxes Y30 haplotypes, purple boxes G (TcI) haplotypes, green boxes CL Brener (TcVI) haplotypes and the pink arrow indicates the Y strain (TcII) cluster. In haplotype names the suffix R indicates the copy detected by direct sequencing of PCR products from genomic DNA while the other indicates the sequence obtained from cloned PCR products. Networks were inferred using SplisTree 4 [24] as described in Material and Methods, with parameters: -lnL = 2550.7314, K = 121, fA = 0.2258, fC = 0.2880, fG = 0.3230, fT = 0.1632, rAC = 1.0000, rAG = 5.7104, rAT = 1.0000, rCG = 1.0000, rCT = 24.9728, rGT = 1.0000, p(Invariants) = 0.5490, alpha(Gamma) = 0.0130.
(TIF)

Figure S2 **Sequences of gp82 carboxy-terminal domain of different** ***T. cruzi*** **strains and the putative gp30.** Shown are the aminoacid sequences deduced from cDNA clones R31 (CL strain), J18 (G strain) and D21 (Y30 strain). Overall, Y30 strain gp30 exhibited sequence identity >50% as compared to CL and G strain gp82. Highlighted are the sequences previously identified as host cell-binding site (p4) and gastric mucin-binding site (p7).
(TIF)

Figure S3 **A) HeLa cells were incubated for 1 h with metacyclic forms, fixed and stained with Giemsa, followed by sequential dehydration as described in experimental procedures.** Internalized metacyclic forms (black arrows) are surrounded by a clear space, distinct from adherent parasites (white arrow). Scale bar = 10 µm. B) Metacyclic forms were incubated with HeLa cells for 1 h, non internalized parasites were washed out, DMEM containing 2% fetal calf serum was added and incubation proceeded for 24 h, upon which the cells were fixed and Giemsa stained. Amastigotes (black arrows) can be visualized. Scale bar = 10 µm.
(TIF)
