# Supplementary figures and images for: Differential Infectivity by the Oral Route of Trypanosoma cruzi Lineages Derived from Y Strain
Source: PLoS Negl Trop Dis. 2012 Oct 4;6(10):e1804. doi: 10.1371/journal.pntd.0001804 (PMC3464286; doi:10.1371/journal.pntd.0001804)

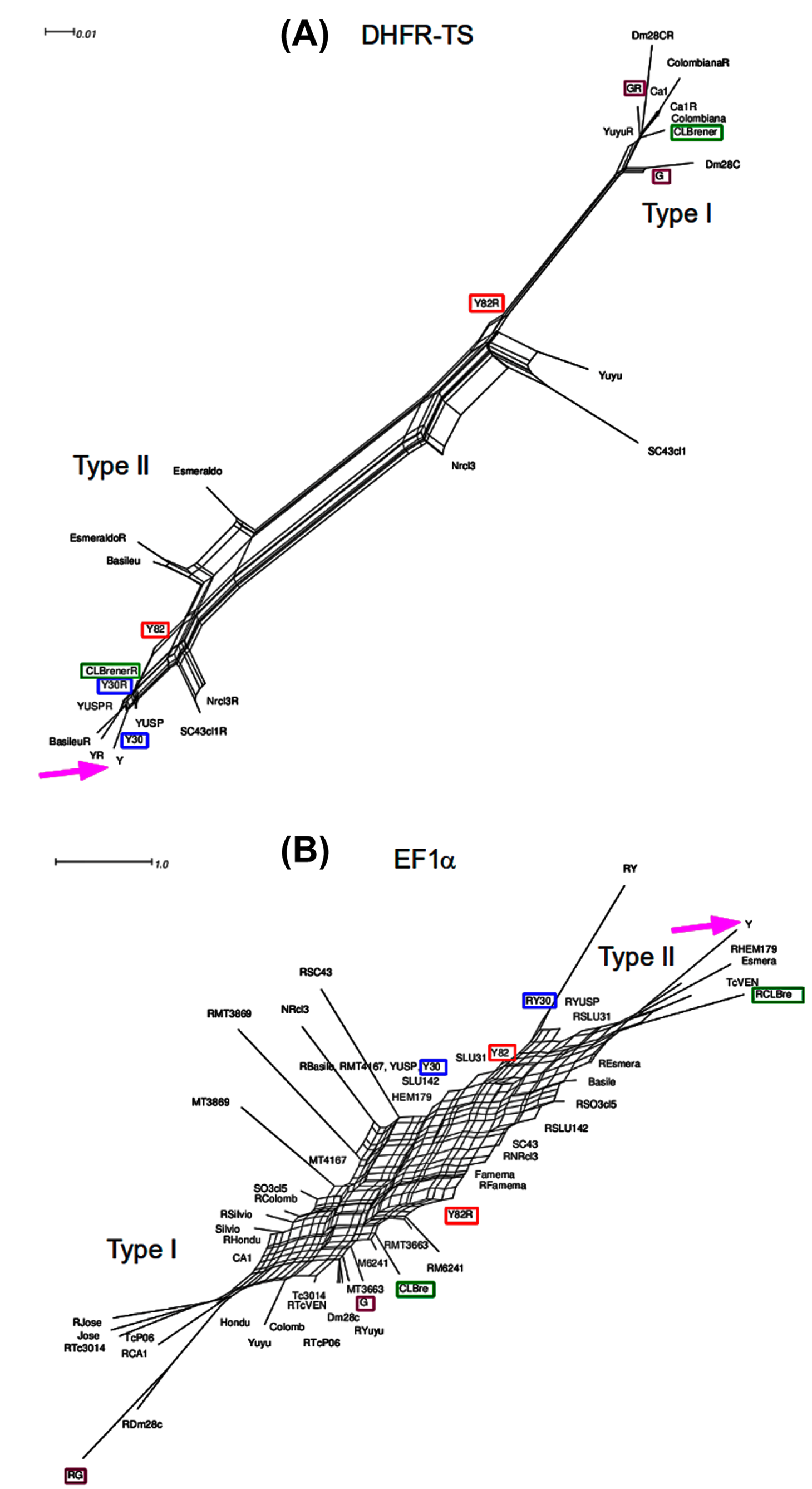

Supplement: Figure S1 — Network genealogies of DHFR-TS and EF1 alpha gene sequences. (A) DHFR-TS and (B) EF1 alpha. The highly reticulated topology in (B) is indicative of intense hybridization, or other gene homogenizing processes, although not informative for grouping and typing. Red boxes indicate Y82 haplotypes, blue boxes Y30 haplotypes, purple boxes G (TcI) haplotypes, green boxes CL Brener (TcVI) haplotypes and the pink arrow indicates the Y strain (TcII) cluster. In haplotype names the suffix R indicates the copy detected by direct sequencing of PCR products from genomic DNA while the other indicates the sequence obtained from cloned PCR products. Networks were inferred using SplisTree 4 [24] as described in Material and Methods, with parameters: -lnL = 2550.7314, K = 121, fA = 0.2258, fC = 0.2880, fG = 0.3230, fT = 0.1632, rAC = 1.0000, rAG = 5.7104, rAT = 1.0000, rCG = 1.0000, rCT = 24.9728, rGT = 1.0000, p(Invariants) = 0.5490, alpha(Gamma) = 0.0130. (TIF) [file pntd.0001804.s001.tif]

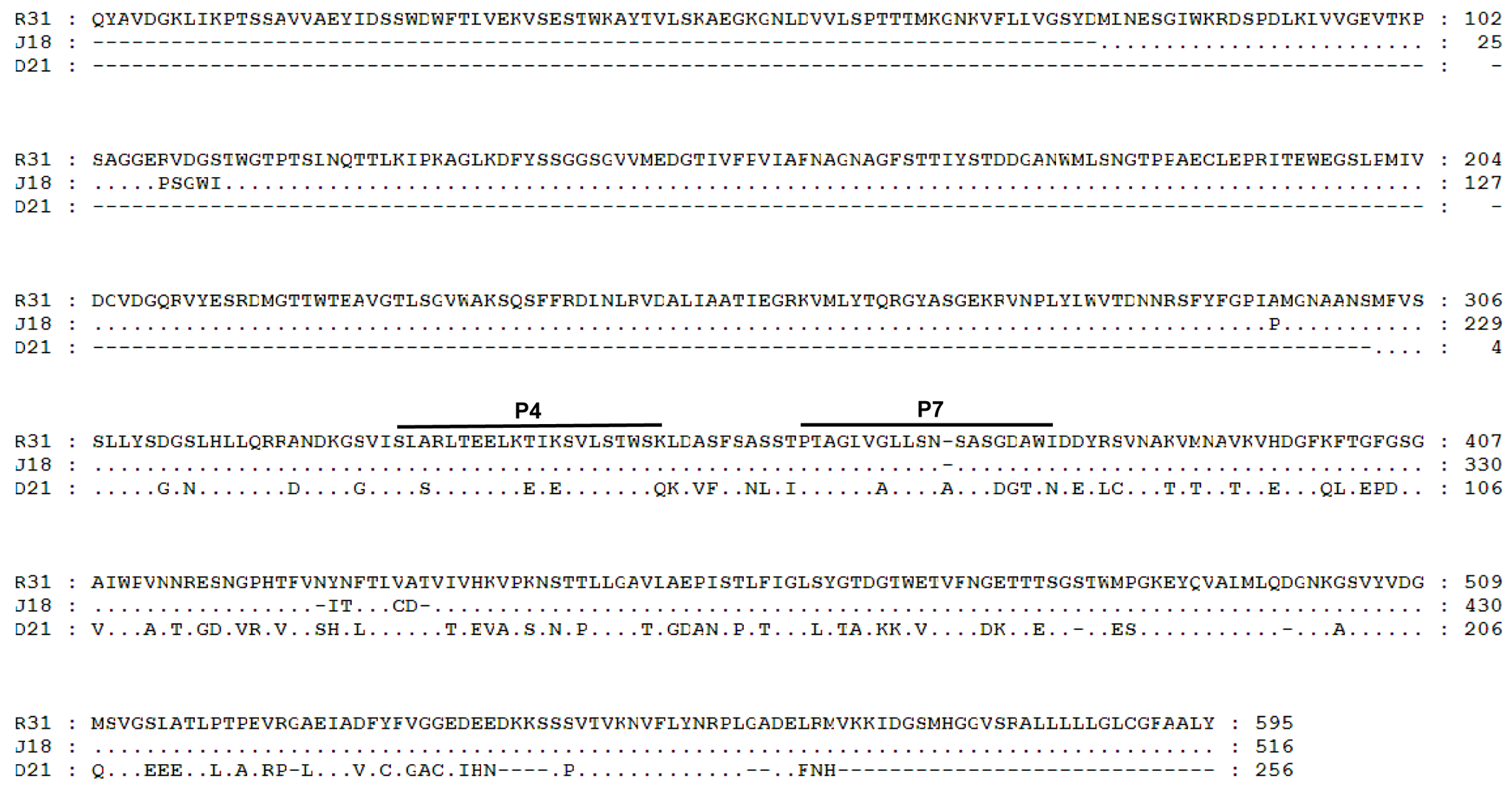

Supplement: Figure S2 — Sequences of gp82 carboxy-terminal domain of different T. cruzi strains and the putative gp30. Shown are the aminoacid sequences deduced from cDNA clones R31 (CL strain), J18 (G strain) and D21 (Y30 strain). Overall, Y30 strain gp30 exhibited sequence identity >50% as compared to CL and G strain gp82. Highlighted are the sequences previously identified as host cell-binding site (p4) and gastric mucin-binding site (p7). (TIF) [file pntd.0001804.s002.tif]

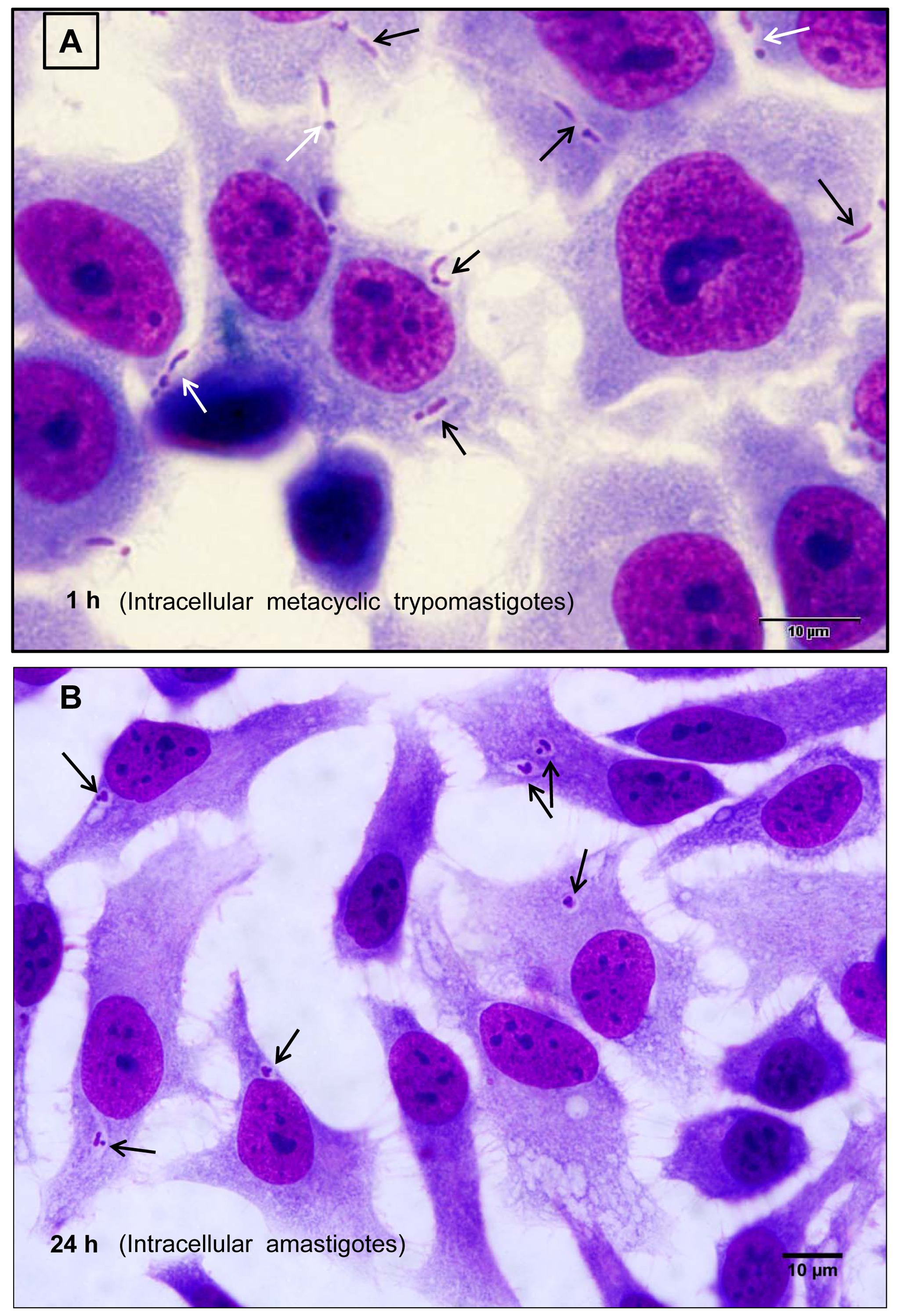

Supplement: Figure S3 — A) HeLa cells were incubated for 1 h with metacyclic forms, fixed and stained with Giemsa, followed by sequential dehydration as described in experimental procedures. Internalized metacyclic forms (black arrows) are surrounded by a clear space, distinct from adherent parasites (white arrow). Scale bar = 10 µm. B) Metacyclic forms were incubated with HeLa cells for 1 h, non internalized parasites were washed out, DMEM containing 2% fetal calf serum was added and incubation proceeded for 24 h, upon which the cells were fixed and Giemsa stained. Amastigotes (black arrows) can be visualized. Scale bar = 10 µm. (TIF) [file pntd.0001804.s003.tif]
